# Evaluation of fabric-based pneumatic actuator enclosure and anchoring configurations in a pediatric soft robotic exosuit

**DOI:** 10.3389/frobt.2024.1302862

**Published:** 2024-10-11

**Authors:** Ipsita Sahin, Mehrnoosh Ayazi, Caio Mucchiani, Jared Dube, Konstantinos Karydis, Elena Kokkoni

**Affiliations:** ^1^ Department of Bioengineering, University of California, Riverside, Riverside, CA, United States; ^2^ Department of Electrical and Computer Engineering, University of California, Riverside, Riverside, CA, United States

**Keywords:** assistive device, upper extremity, wearable design, kinematics, soft robotics, infant

## Abstract

**Introduction:**

Soft robotics play an increasing role in the development of exosuits that assist, and in some cases enhance human motion. While most existing efforts have focused on the adult population, devices targeting infants are on the rise. This work investigated how different configurations pertaining to fabric-based pneumatic shoulder and elbow actuator embedding on the passive substrate of an exosuit for pediatric upper extremity motion assistance can affect key performance metrics.

**Methods:**

The configurations varied based on actuator anchoring points onto the substrate and the type of fabric used to fabricate the enclosures housing the actuators. Shoulder adduction/abduction and elbow flexion/extension were treated separately. Two different variants (for each case) of similar but distinct actuators were considered. The employed metrics were grouped into two categories; reachable workspace, which includes joint range of motion and end-effector path length; and motion smoothness, which includes end-effector path straightness index and jerk. The former category aimed to capture first-order terms (i.e., rotations and displacements) that capture overall gross motion, while the latter category aimed to shed light on differential terms that correlate with the quality of the attained motion. Extensive experimentation was conducted for each individual considered configuration, and statistical analyses were used to establish distinctive strengths, weaknesses, and trade-offs among those configurations.

**Results:**

The main findings from experiments confirm that the performance of the actuators can be significantly impacted by variations in the anchoring and fabric properties of the enclosures while establishing interesting trade-offs. Specifically, the most appropriate anchoring point was not necessarily the same for all actuator variants. In addition, highly stretchable fabrics not only maintained but even enhanced actuator capabilities, in comparison to the less stretchable materials which turned out to hinder actuator performance.

**Conclusion:**

The established trade-offs can serve as guiding principles for other researchers and practitioners developing upper extremity exosuits.

## 1 Introduction

Upper Extremity (UE) wearable assistive and rehabilitation devices for the adult population have witnessed significant advancements in recent years (Majidi Fard Vatan et al., 2021). Examples range from rigid (Gopura et al., 2011; Balasubramanian and He, 2012; Rahman et al., 2015) and cable-driven exoskeletons (Gaponov et al., 2017; Xiao et al., 2017; Herbin and Pajor, 2021) to soft wearable devices (Polygerinos et al., 2015; Nguyen and Zhang, 2020; Zhou et al., 2024). Soft wearable devices, in particular, have been increasingly employing pneumatic actuators owing to the latter’s key features: low mass, inherent safety (i.e., low injury risk from malfunction), high power-to-weight ratio, affordability, and ease of construction (Maeder-York et al., 2014; Polygerinos et al., 2015; Yap et al., 2017; Nguyen et al., 2019; Majidi Fard Vatan et al., 2021). Despite the abundance of UE wearable devices for adults, devices tailored to the specific needs of pediatric populations remain comparatively limited (Arnold et al., 2020). This is concerning given the potential of assistive technology to positively impact motor function in these populations (Henderson et al., 2008; Guerette et al., 2013).

The lack of devices is especially prominent for those under the age of 2 years (Christy et al., 2016; Arnold et al., 2020). The unique characteristics of this population (such as rapid changes in their growth and learning as well as the complexity of the activities they are engaged in) pose challenges in technology design and implementation (Huelke, 1998; Arnold et al., 2020). For example, the kinematic parameters of reaching, one of the most important motor milestones involving the UEs (Gerber et al., 2010), undergo constant changes during the first 2 years of life, and reach adult-like levels only after this period (Konczak and Dichgans, 1997). Hence, designing an assistive device for this population requires careful consideration of the aforementioned challenges since they can affect both the device’s efficacy as well as its safety and usability (Lobo et al., 2019; Scherer et al., 2005). Recently, there has been a push to develop wearable technology for UE movement assistance for the infant population. Some notable examples include the rigid passive exoskeleton P-WREX (Babik et al., 2016), garment-based Playskin Lift (Lobo et al., 2016), and soft robotic exosuit prototypes that encompass silicone-based (Kokkoni et al., 2020) and fabric-based (Sahin et al., 2022; 2023; Mucchiani et al., 2022; 2023) pneumatic actuators.

The functionality of wearable devices employing soft actuators depends on several parameters. Crucially, most existing efforts evaluate the employed actuators’ performance in isolation from the overall device. There has been less effort toward understanding how the performance of those actuators may be affected by textile integration, or how the placement of those actuators around the joints may affect motion generation (Zannat et al., 2023). Notably, the optimal placement of an actuator at a specific joint to achieve the desired motion is highly debated (Kokubu et al., 2024; Yap et al., 2016; Wehner et al., 2013). It has been suggested that, unlike rigid devices, soft actuators may handle imprecise placement about a joint (Yap et al., 2017; Yap et al., 2016) since they can passively absorb the effects of misalignment of the axis of rotation and unexpected loads (Shiota et al., 2019). However, it has been noted that the anchoring position significantly influences joint kinematics and kinetics (Kokubu et al., 2024; Wehner et al., 2013). Proper anchoring influences the transfer of forces to the body as well as stabilizes the interaction between the body and the wearable device (Samper-Escudero et al., 2020; Bae et al., 2018). Hence, applying appropriate pressure levels at the correct points is essential for providing assistance to the body (Kokubu et al., 2024). Excessive pressure, on the other hand, can hinder natural movement of the body, cause discomfort (Kokubu et al., 2024), skin thinning (Mak et al., 2010), blood circulation disorders, and injuries (Schiele and Van der Helm, 2009; Mayrovitz and Sims, 2003; Bringard et al., 2006), thus limiting the device’s adoption (Bright and Coventry, 2013).

Another critical parameter affecting the wearable device’s performance relates to the fabrics and their properties (Zannat et al., 2023; Piao et al., 2023). This concerns the use of fabrics both as the main building material for the actuators and as the substrate on top of (and/or within) which an actuator (of any type) is anchored. For example, soft actuators made of elastomeric fabrics of high tensile strength (e.g., thermoplastic polyurethane [TPU] films (Nguyen and Zhang, 2020)) yield several advantages over their silicone counterparts; they can be built faster and at a lower cost, are considerably less bulky, and can generate higher forces (Suulker et al., 2022; Agarwal et al., 2016; Sahin et al., 2022). However, different types of fabrics have complex microstructures which can lead to very distinctive and often diverging properties all while modeling the behavior of the composites is already a difficult task (Cappello et al., 2018). Even fabrics sharing the same name can exhibit variations in composition and properties. Further, fabrics with similar compositions may differ in texture, elasticity, tensile strength, and other characteristics due to factors such as thread type and knitting process (Zannat et al., 2023). Thus, the choice of fabric type can significantly influence the actuator’s performance, due to their wide range of stretch and strain properties (Cappello et al., 2018; Zhang et al., 2023).

In this paper, we conducted a systematic examination to understand the impact of soft actuator integration within an UE exosuit designed for use by the infant population. First, we fabricated soft pneumatic actuators of different sizes and shapes which have a low profile and can generate a sufficient range of motion (ROM) and force (Sahin et al., 2022; 2023). Then, we investigated how the actuator’s anchoring around each joint affects key motion characteristics. Lastly, we tested a range of different fabrics used to create the enclosures (i.e., pockets) within which the actuators are housed. We hypothesized that embedding the actuators at different anchoring points (Kokubu et al., 2024; Wehner et al., 2013) as well as using different types of fabric for the detachable pockets (Cappello et al., 2018; Zhang et al., 2023; Adams and Keyserling, 1995) would vary the performance of the actuators. In the following sections, we present the methodology employed for fabric selection, actuator integration techniques, and evaluation of the performance and functionality of the embedded actuators. Our findings can unfold potential applications and implications, fostering progress in developing UE assistive wearable exosuits for young populations.

## 2 Methods

### 2.1 Experimental design

A series of experiments were carried out to determine how two key features, which pertain to actuator embedding on the passive substrate^1^ of the exosuit prototype, affect UE kinematics on a physical model. The physical model was scaled to closely match the 50th percentile of a 12-month-old infant’s upper body. Thus, based on related anthropometrics literature (Fryar et al., 2021; Edmond et al., 2020; Schneider and Zernicke, 1992), the upper arm and forearm weigh ∼0.20 kg and ∼0.18 kg, and measure a length of 15 cm and 11 cm, respectively. The two features included i) the *positioning/anchoring of the actuators* on the substrate, and ii) the *fabric properties of detachable pockets* containing the actuators.

A specific class of actuators was considered in this work. The actuators feature one or multiple (connected in-series) cells of different shapes (square/circular profile) and sizes that elongate/shorten based on the appropriate pneumatic input. Two variants of actuators for each joint were included in the experiments conducted herein. At the shoulder joint, there were two rectangular actuator variants based on the number of air cells (1-cell and 2-cell). At the elbow joint, there were two 10-cell bellow actuator variants based on cell shape (square and circular; side length/diameter fixed at 3 cm). The shoulder actuators work with positive pressure whereas the elbow actuators are vacuum-powered. This design selection was based on prior work (Sahin et al., 2022; Sahin et al., 2023) which determined their suitability in the context of infant wearable exosuits, and, crucially, assessed the performance of the specific actuator variants employed herein while systematically altering their aforementioned features. In summary, the 1-cell rectangular actuator demonstrated appropriate force generation and support for shoulder abduction/adduction, while the 2-cell actuator exhibited higher reproducibility (Sahin et al., 2022). Further, the 10-cell circular actuator achieved higher ROM during elbow flexion and extension, while the square actuator produced smoother end-effector motion (Sahin et al., 2023).

All actuator variants were made of flexible and lightweight TPU fabric (Oxford 200D heat-sealable coated fabric of 0.20 mm thickness) following the steps outlined in (Sahin et al., 2022; 2023) and were checked for air leakages prior to the experiments on the physical model. Fabrication time for each variant was between 0.5 and 1.5 h. Each actuator provided 1-DoF assistance at each joint (shoulder abduction/adduction and elbow flexion/extension) while not obstructing the remaining DoFs at that joint. Actuator inflation/deflation was regulated through an off-body pneumatic control board (see Section 2.2). The actuators were attached to the physical model at different anchoring points to determine the best performance (Section 2.1.1). Then, at the down-selected anchoring point, these actuators were embedded within removable pockets that were custom-made from different materials, and their performance was again evaluated (Section 2.1.2). Detailed information is provided next.

#### 2.1.1 Positioning/anchoring of actuators

**FIGURE 1:**
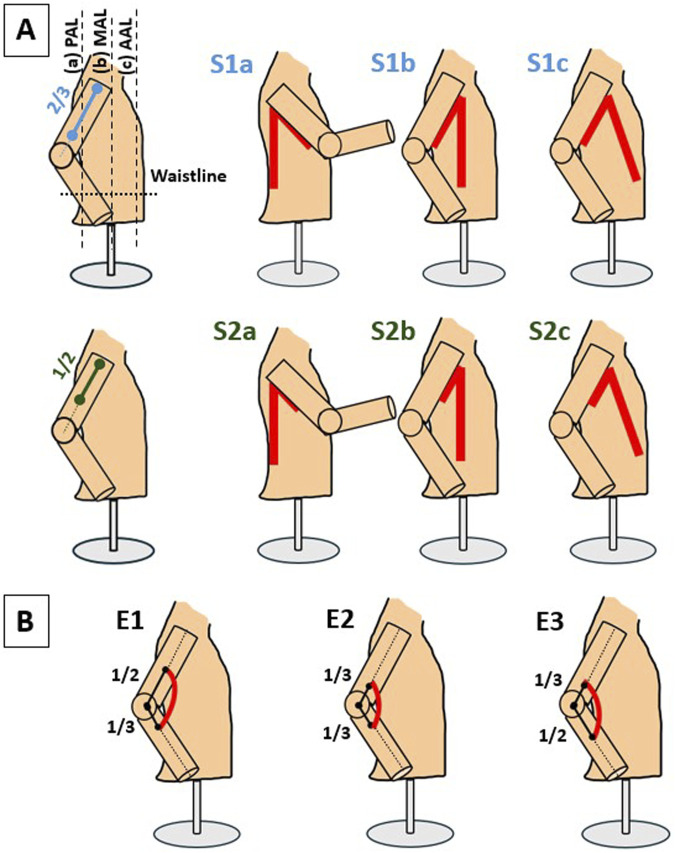
**(A)** Six actuator placement configurations were considered for the shoulder joint by varying the attachment points on the UA and waistline. On the UA, two attachment points were selected. At an offset distance of two-thirds the segment length from its proximal end (S1 - top row), and at the midpoint of the segment (S2 - bottom row). On the waistline, three attachment points **(A–C)** were considered intersecting along the posterior axillary line (PAL), mid axillary line (MAL), and anterior axillary line (AAL) respectively. **(B)** A total of three configurations were considered for the elbow joint. A symmetric one resulting from attachment points at an offset distance of one-third each segment’s length from the elbow joint center (E2), and two asymmetric ones with varying distance offset (at one-third and one-half from the elbow joint center) for each involved segment (E1 and E3). Actuators are depicted in red.

The first series of experiments aimed at understanding how the different actuator attachment points affect the physical model’s arm motion. At the shoulder joint, the two ends of the actuators were attached (via straps) to the upper arm (UA) and the waistline respectively, leading to a total of six distinct configurations (Figure 1A). On the UA, two different attachment points were considered; one at an offset distance of two-thirds the segment length from its proximal end (Figure 1A [top row]), and another at the midpoint of the segment (Figure 1A [bottom row]). On the waistline, three attachment points were considered intersecting along the posterior axillary line (PAL), mid axillary line (MAL), and anterior axillary line (AAL).

At the elbow joint, the attachment points of the actuators on the UA and forearm were selected based on the distance of their ends from the elbow joint center (Figure 1B). Initially, the possibility of placing the actuators at the posterior/dorsal side of the arm was explored, similar to Kokkoni et al. (2020). However, this placement encountered a challenge as the elbow actuator variants in Sahin et al. (2023) struggled to induce the desired flexion of the elbow joint effectively. Consequently, a strategic decision was made to relocate the actuator placement to the anterior/ventral side of the arm (Figure 1B). This adjustment aimed at optimizing the actuator’s ability to facilitate the desired motion about the elbow joint while aligning with the functional objectives of the wearable device. Three different configurations were assessed by varying the attachment points on the UA and forearm (Figure 1B). One (symmetric) configuration (E2) resulting from an equal number of cells extending at an offset distance of one-third each segment’s length from the elbow joint center. The other two (asymmetric) configurations (E1, E3) resulting from varying the attachment offset on each segment (at one-third and one-half distance from the elbow joint center).

Note that the pressure applied by the straps may affect the performance of the actuators as well as increase variability. Therefore, to ensure consistency of actuator placement across experiments, specific markings were used to highlight where the strap had to be placed. The actuator anchoring points that yielded the most effective motion at the elbow and shoulder joints were down-selected and used in conjunction with the detachable pockets in the next series of experiments.

#### 2.1.2 Fabric properties of actuator enclosure

The second series of experiments aimed at understanding how embedding the (down-selected) actuators into the detachable pockets may affect the actuators’ performance. As a direct outcome of the first series of experiments on anchoring points (see Section 3.1), the shoulder and the elbow actuators were attached in the S1b and E2 configurations, respectively (Figure 1). Four types of fabric were used to fabricate the enclosures: nylon, jersey, denim, and polyester. Their properties are listed in Table 1.

**TABLE 1:** Properties of the tested fabrics that were used to fabricate the actuator enclosures.

Fabric type	Composition (%)	Tensile strength^*^ (x10^6^ N/m^2^)	Expansion^*^ (%)	Thickness (mm)	Price ($/yd)
Denim	100% cotton	18.12±5.30	11.76±1.67	0.80	20.99
Jersey	95% cotton and 5% spandex	11.83±4.50	50.15±22.06	0.50	12.99
Nylon	82% nylon and 18% spandex	30.93±4.87	40.41±10.80	0.60	17.99
Polyester	65% polyester and 35% cotton	11.34±1.72	9.77±0.78	0.10	4.99

^*^ Values were determined based on tensile testing using a strain-stress apparatus.

Fabrics were manually trimmed to the desired dimensions using scissors, and their edges were carefully folded and stitched together using a sewing machine. The dimensions of the shoulder and elbow actuators were 6×20 cm and 3×3×15 cm respectively (Sahin et al., 2022; Sahin et al., 2023). The dimensions of the trimmed fabric for the detachable pockets were 16×21 cm and 25×16 cm for the shoulder and elbow actuator, respectively. To house the shoulder actuator within the pockets, a fabric size of at least 12×20 cm is necessary, whereas for the elbow actuator a minimum of 21×15 cm area is required (to cover the circumference of the arm [12 cm] and the perimeter of the actuator [9 cm]). The additional length and width of the fabric were allocated for folding, stitching, and attaching hook-and-loop Velcro fasteners.

**FIGURE 2:**
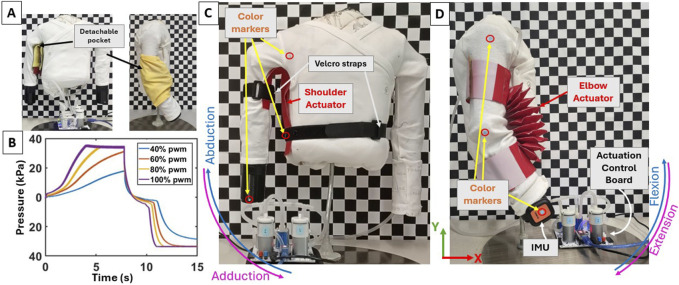
**(A)** Fabric pockets can be attached and detached directly to the exosuit using hook-and-loop Velcro fasteners. **(B)** Evolution of the pressure buildup over time inside the 1-cell shoulder actuator at different %PWM. The selected 100% PWM is the quickest to inflate and deflate the actuator. **(C)** Physical prototype with a shoulder actuator attached as per the S2b configuration. Velcro straps are used to hold the actuator on ends (which are not inflatable) in place. **(D)** Snapshot from elbow flexion/extension experiments. Here, the actuator is placed on the ventral side of the arm as per the E2 configuration.

As in the first round of experiments, attachment points of the pockets on the substrate were carefully labeled to ensure consistent placement. The Velcro hooks and loops were attached to the pockets and the substrate, respectively, to enable a direct way for pocket detachment and re-attachment (Figure 2A). Such design approach allows for easy actuator repair and/or replacement without the need to take off the entire exosuit; instead, only the pocket needs to be removed and fixed. We note that during preliminary experimentation, we also explored the use of snap buttons similar to Golgouneh et al. (2021) as an alternative way of attachment which, compared to our selected method, demonstrated two key limitations. First, the pocket was attached to the substrate based on a few distinctive points, which led to undesired actuator relative motion and/or deformation during inflation/deflation. Second, snap buttons require greater force to attach to each other, as compared to using Velcro, which might increase the pressure exerting on the body.

### 2.2 System operation protocol

**FIGURE 3:**
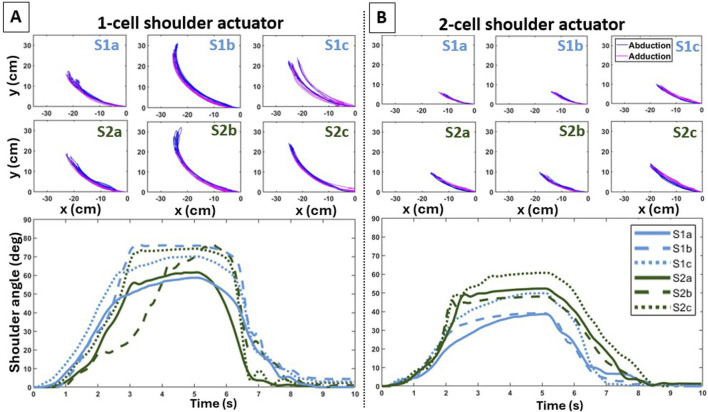
Individual trajectories of the end-effector (top panels) and average curves in shoulder joint angle (bottom panels) during inflation (abduction) and deflation (adduction) across the six configurations for the 1-cell **(A)** and 2-cell **(B)** shoulder actuators.

The inflation and deflation of the actuators were regulated through an off-body pneumatic control board (Programmable-Air hardware kit). The board weighs 0.35 kg and incorporates two compressor/vacuum pumps and three pneumatic valves to precisely manage airflow at 2 L per minute during both inflation and deflation. The board can generate pressure within the range [−50,50] kPa. The pumps modulate air pressure rate via the duty cycle which ranges from 0% to 100%. It is worth noting that while the pump may activate at approximately 20% duty cycle, lower duty cycles result in a longer inflation/deflation duration (Figure 2B). Therefore, we operated the actuation control board at 100% duty cycle as it offered the quickest inflation/deflation. Note that the duration of typically developing infants’ full-reaching actions is less than 2 seconds (Zhou and Smith, 2021). We thus aimed for actuator full inflation and deflation times to be as short as possible. To ensure we achieve full inflation (and deflation) of the actuators using the selected pneumatic board at 100% pump duty cycle, we aimed for an operation duration of 5 seconds; nevertheless, full inflation (and deflation) can be achieved within 2 to 3 seconds, as shown in the Results section (Figures 3, 5). An Arduino Nano (ATMega328P) single-board computer was used to interface the Programmable-Air board with a workstation (e.g., for data logging and analysis). The Programmable-Air board receives power from a 12V adapter and is equipped with a pressure sensor (SMPP-03). Additionally, after achieving full inflation, an automatic cutoff mechanism engages when a certain internal pressure threshold (set at approximately 34 kPa) is reached, preventing potential leakage and safeguarding the actuators from damage. Each actuator underwent a series of 10 trials per condition of the experiment (i.e., different anchoring positions and fabric properties).

### 2.3 Data analysis

#### 2.3.1 Evaluation metrics

Kinematic data were obtained from video recordings and Inertial Measurement Units (IMUs; XSens DOT, Movella Inc.) at a sampling rate of 30 fps and 60 Hz, respectively. Video recordings provided information on the 2D positions of the shoulder and elbow joints as well as the end-effector (distal end of forearm). These positions were indicated by color markers (0.10 cm) placed on the arm, as shown in Figures 2C, D, and were extracted using DLTdv8 (Hedrick, 2008), a MATLAB (MathWorks Inc.) tool designed for video file digitization. The IMUs provided information on the acceleration of the end-effector.

The variables considered herein were the joint ROM (for both shoulder and elbow joints), as well as end-effector path length, straightness index (SI), jerk, and Lock-Step Euclidean Distance (LSED). This selection is in accordance with prior related work (Zhang et al., 2008; Kokkoni et al., 2020; Sahin et al., 2022; Sahin et al., 2023). Shoulder and elbow joint ROM were computed indirectly, by calculating the relative angles between the torso and upper arm line segments, and upper arm and forearm segments, respectively. Those lines were attained by tracking the position of the fiducial markers placed in each segment. In this work, configurations affording larger ROM were sought after. The end-effector path length (i.e., total distance traveled) was computed directly from the end-effector position data. In the analysis that follows, the ROM and the end-effector path length were grouped under the category “Reachable Workspace,” considering that they both pertain to first-order physical quantities (i.e., rotations and displacements). It is worth noting that a longer path length does not necessarily correlate with larger ROM as it may also indicate the presence of non-smooth and superfluous motion (for instance back-and-forth arm sway motion). For this reason, it was also important to infer end-effector motion smoothness by calculating SI and jerk. The SI is the (dimensionless) ratio of the actual path length to the vector norm between the initial and final position points. The attained motion has a better adherence to the straight-line motion path (i.e., fewer instances of back-and-forth sway motion) as SI →1 ; hence, configurations with SI values close to one were sought after. To quantify the actual trajectory smoothness, it is crucial to also employ higher-order derivative terms. Jerk (i.e., the rate at which acceleration changes with respect to time) was used here and was computed via direct differentiation of IMU data placed on the end-effector (Figure 2D). The Root Mean Square (RMS) amplitude of jerk was then computed. Low RMS values for jerk indicate smoother paths, which is a desirable trait for the considered configurations. In the analysis that follows, the end-effector path SI and jerk were grouped under the category “Motion Smoothness.” Lastly, the LSED (Tao et al., 2021) was computed between the trajectories attained with each considered configuration to assess their variability, with lower values denoting less variability. All the aforementioned computations were performed in MATLAB.

#### 2.3.2 Statistical analysis

Non-parametric tests were performed to assess the potential effect of varying the anchoring points and fabric types on the reachable workspace and motion smoothness (violation of normality was confirmed with the Kolmogorov-Smirnov test). To assess changes due to varying the attachment points on the UA (S1, S2) and the waistline (PAL, MAL, AAL), Mann-Whitney U and Kruskal–Wallis H tests were conducted, respectively. Accordingly, Kruskal–Wallis H tests were performed to determine group differences across the elbow actuator attachment configurations (E1, E2, and E3). To assess changes in the reachable workspace and motion smoothness due to the different fabric types used for the detachable pockets (nylon, jersey, denim, polyester, and no-fabric), Kruskal–Wallis H tests were conducted. The significance level at 0.05 was Bonferroni-adjusted to account for multiple comparisons. The aforementioned statistical approach was followed for all shoulder and elbow actuator variants. Statistical analyses were conducted with SPSS v.27.

## 3 Results

### 3.1 Role of positioning/anchoring points

#### 3.1.1 Effects on shoulder abduction/adduction

Overall, distinct attachment points affected the reachable workspace and motion smoothness differently for each shoulder actuator variant. The trajectories of the end-effector’s 2D position in the frontal plane visually portray the presence of motion variability created by each shoulder actuator variant across the different configurations (Figure 3 [top panels]). The LSED values shown in Table 2 confirm the presence of variability, with lower values indicating less variability for the 2-cell actuator. Additionally, the bottom panels of Figure 3 illustrate the shoulder joint angle over time for the different configurations, showing that, overall, the 2-cell actuator exhibited smaller ROM than the 1-cell actuator (Figure 3 [bottom panels]). Detailed information is provided in the following sections.

**TABLE 2:** LSED values for shoulder abduction and adduction trajectories across the different configurations.

Configurations	**LSED** (mean ± SD) (cm)			
	Abduction		Adduction	
	(A) 1-cell	(B) 2-cell	(A) 1-cell	(B) 2-cell
S1a	2.09±0.10	0.92±0.12	1.86±0.06	0.83±0.11
S1b	1.23±0.04	0.47±0.05	1.49±0.08	0.60±0.12
S1c	1.57±0.06	0.74±0.14	1.05±0.06	1.22±0.30
S2a	1.33±0.06	0.64±0.03	1.27±0.08	0.73±0.05
S2b	3.59±0.52	0.91±0.07	0.89±0.05	1.02±0.08
S2c	1.17±0.19	1.44±0.19	0.55±0.03	0.99±0.14

##### 3.1.1.1 Reachable workspace

**FIGURE 4:**
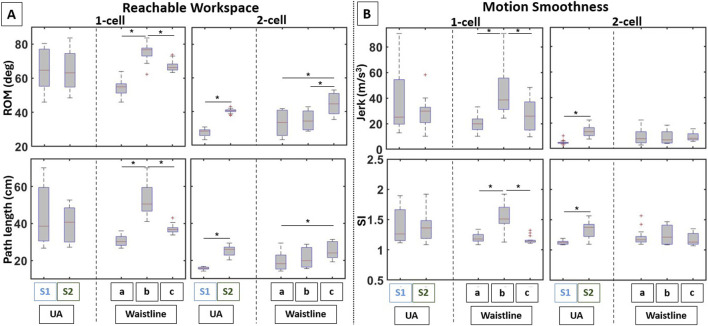
Boxplots of the computed variables for the shoulder actuator variants in terms of **(A)** reachable workspace and **(B)** motion smoothness. Results show significant performance differences owing to different actuator attachment points.

Varying the attachment points of the 1-cell actuator on the UA did not significantly affect shoulder ROM ( U=417 , p=0.626 ) or end-effector path length ( U=427 , p=0.734 ). However, varying the attachment points on the waistline had a significant effect on both ROM ( $\chi^{2}\left(2\right)=48.289$ , p<0.001 ) and path length ( $\chi^{2}\left(2\right)=50.052$ , p<0.001 ). Post-hoc analyses revealed that ROM was significantly greater when the actuator was attached along the MAL (75.44 ± 4.60^0^) as compared to the AAL (67.14 ± 2.97^0^, p=0.006 ) and the PAL (54.40 ± 4.20^0^, p<0.001 ) (Figure 4A [top row]). Similarly, the path length was significantly larger when the actuator was attached along the MAL (52.49 ± 7.99 cm) as compared to the AAL (36.91 ± 2.26 cm, p=0.001 ) and PAL (30.57 ± 2.88 cm, p<0.001 ) (Figure 4A [bottom row]).

The following observations were noted for the 2-cell actuator. Varying the attachment points on the UA significantly affected shoulder ROM ( U=872.50 , p<0.001 ), with greater values observed (Figure 4A [top row]) in the S2 attachment (43.87 ± 5.08^0^) in comparison to S1 (31.34 ± 5.54^0^). Similarly, varying the attachment points on the waistline had a significant effect on shoulder ROM ( $\chi^{2}\left(2\right)=13.040$ , p=0.001 ), with greater values observed when the actuator was attached along the AAL (44.65 ± 6.37^0^) as compared to the MAL (34.84 ± 5.39^0^, p=0.015 ) and the PAL (33.33 ± 7.58^0^, p=0.002 ). Further, varying the attachment points on the UA significantly affected the end-effector’s path length ( U=880.50 , p<0.001 ), with the S2 attachment resulting in a larger path length (26.40 ± 3.24 cm) than S1 (17.54 ± 2.81 cm). Varying the attachment points on the waistline also affected path length ( $\chi^{2}\left(2\right)=11.650$ , p=0.003 ). Post-hoc comparisons revealed a larger path length when the actuator attachment point was along the AAL (25.22 ± 4.37 cm, p<0.001 ) as compared to PAL (19.30 ± 4.63 cm, p=0.002 ) but not MAL (21.37 ± 5.52 cm, p=0.256 ).

##### 3.1.1.2 Motion smoothness

Varying the 1-cell actuator’s attachment points on the UA did not have a significant effect on the end-effector’s jerk ( U=498.50 , p=0.473 ). In contrast, varying the attachment points on the waistline did have an effect ( $\chi^{2}\left(2\right)=24.734$ , p<0.001 ), with higher jerk values (i.e., less smooth motion) observed (Figure 4B [top row]) when the actuator attachment point on the waistline was along the MAL (44.47 ± 17.75 m^-3^) as compared to the AAL (27.39 ± 12.01 m^-3^, p=0.007 ) and PAL (20.17 ± 6.47 m^-3^, p<0.001 ). Similarly, varying the attachment points on the UA did not have a significant effect on the SI ( U=432.00 , p=0.790 ); however, varying the attachment points on the waistline did have an effect ( $\chi^{2}\left(2\right)=31.785$ , p<0.001 ). Post-hoc comparisons revealed that the SI was significantly greater (i.e., less smooth motion) when the actuator was attached along the MAL (1.54 ± 0.21) as compared to the AAL (1.16 ± 0.06, p<0.001 ) and PAL (1.20 ± 0.07, p<0.001 ) (Figure 4B [bottom row]).

Unlike the 1-cell actuator, the 2-cell actuator’s attachments on the UA and waistline exhibited opposite changes in the variables. Varying the attachment points on the UA produced a significant effect on jerk ( U=891.00 , p<0.001 ), with greater jerk values observed (Figure 4B [top row]) in the S2 attachment (12.80 ± 3.89 m^-3^) as compared to the S1 (5.38 ± 1.55 m^-3^). Varying the actuator attachment points on the waistline did not significantly affect jerk ( $\chi^{2}\left(2\right)=0.964$ , p=0.618 ). Further, varying the attachment points on the UA significantly affected SI ( U=778.00 , p<0.001 ), with a greater SI observed (Figure 4B [bottom row]) for S2 (1.30 ± 0.13) in comparison to S1 (1.12 ± 0.03). No significant difference in SI was observed for the attachment points on the waistline ( $\chi^{2}\left(2\right)=2.547$ , p=0.280 ).

#### 3.1.2 Effects on elbow flexion/extension

**FIGURE 5:**
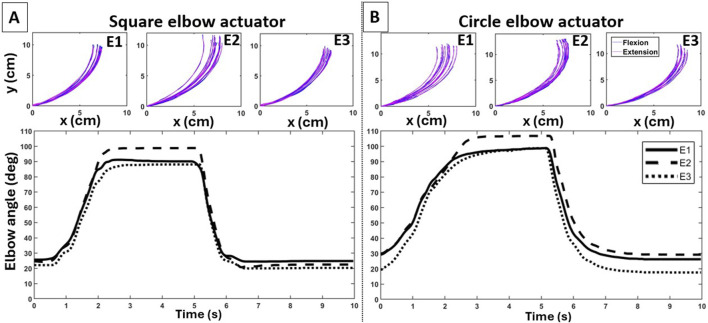
Individual trajectories of the end-effector (top panels) and average curves in elbow joint angle (bottom panels) during deflation (flexion) and inflation (extension) across the three configurations for the square **(A)** and circular **(B)** elbow actuators.

Overall, attaching the elbow actuator variants at different points on the UA and forearm affected arm motion, with the symmetric configuration contributing to a greater reachable workspace but not smoothness. Observing the evolution of the 2D position of the end-effector on the sagittal plane highlights the motion variability created by each elbow actuator variant for the different configurations (Figure 5 [top panels]). The LSED values reported in Table 3 support that greater motion variability across the trials was observed during the elbow extension phase. Lastly, the bottom panels of Figure 5 illustrate the elbow joint angle over time for the different configurations.

**TABLE 3:** LSED values for elbow flexion and extension trajectories across the different configurations.

Configuration	**LSED** (mean ± SD) (cm)			
	Flexion		Extension	
	Square	Circular	Square	Circular
E1	0.68±0.11	1.17±0.07	3.80±0.17	3.05±0.15
E2	0.87±0.07	0.71±0.07	5.69±0.72	3.21±0.21
E3	0.54±0.03	7.72±2.54	4.68±0.55	3.06±0.14

##### 3.1.2.1 Reachable workspace

**FIGURE 6:**
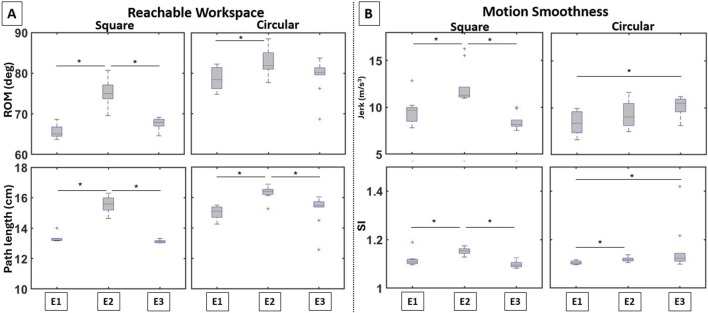
Boxplots of the computed variables for the elbow actuator variants in terms of **(A)** reachable workspace and **(B)** motion smoothness. Notable differences between the symmetric and asymmetric elbow actuator placements can be observed.

Significant differences in elbow ROM were noted across the various configurations (Figure 6A [top row]) for both the square ( $\chi^{2}\left(2\right)=21.920$ , p<0.001 ) and the circular ( $\chi^{2}\left(2\right)=6.259$ , p=0.044 ) actuators. Specifically, ROM was significantly greater when the square actuator was attached in E2 (75.22 ± 3.75^0^) as compared to E1 (65.65 ± 1.67^0^, p<0.001 ) and E3 (67.51 ± 1.63^0^, p=0.008 ) configurations. For the circular actuator, ROM was significantly greater when it was attached in E2 (82.62 ± 3.11^0^) as compared to E1 (78.64 ± 2.89^0^, p=0.047 ) but not E3 (79.42 ± 4.32^0^, p=0.226 ). Additionally, significant differences were found in end-effector path length across the different configurations for both the square ( $\chi^{2}\left(2\right)=23.056$ , p<0.001 ) and circular ( $\chi^{2}\left(2\right)=16.759$ , p<0.001 ) actuators. Post-hoc comparisons for the square actuator revealed a significantly larger path length in the E2 (15.55 ± 0.54 cm) as compared to the E1 (13.32 ± 0.25 cm, p=0.013 ) and E3 (13.13 ± 0.10 cm, p<0.001 ) configurations. Lastly, path length was significantly larger in E2 (16.32 ± 0.43 cm), compared to the E1 (15.00 ± 0.44 cm, p<0.001 ) and E3 (15.21 ± 1.01 cm, p=0.021 ) for the circular actuator.

##### 3.1.2.2 Motion smoothness

Significant differences in jerk were observed across the different configurations for both the square ( $\chi^{2}\left(2\right)=18.866$ , p<0.001 ) and circular ( $\chi^{2}\left(2\right)=8.168$ , p=0.017 ) actuators (Figure 6B [top row]). Jerk was found to be significantly greater in the E2 configuration (12.28 ± 1.94 m^-3^), in comparison to E1 (9.67 ± 1.37 m^-3^, p=0.020 ) and E3 (8.48 ± 0.83 m^-3^, p<0.001 ) for the square actuator. For the circular actuator, jerk was significantly greater for E3 (10.23 ± 0.95 m^-3^) as compared to E1 (8.36 ± 1.19 m^-3^, p=0.013 ) but not E2 (9.35 ± 1.50 m^-3^, p=0.443 ). Additionally, significant differences were noted in the SI due to the configurations for both the square ( $\chi^{2}\left(2\right)=17.828$ , p<0.001 ) and circular ( $\chi^{2}\left(2\right)=10.514$ , p=0.005 ) actuators. For the square actuator, the SI was significantly greater in E2 (1.15 ± 0.01) as compared to E1 (1.12 ± 0.03, p=0.019 ) and E3 (1.10 ± 0.02, p<0.001 ). But for the circular actuator, the SI was significantly lower in the E1 (1.10 ± 0.01) as compared to the E2 (1.12 ± 0.01, p=0.048 ) and E3 (1.16 ± 0.06, p=0.006 ) configurations.

### 3.2 Role of fabric properties for the actuator enclosure

#### 3.2.1 Effects on shoulder abduction/adduction

**FIGURE 7:**
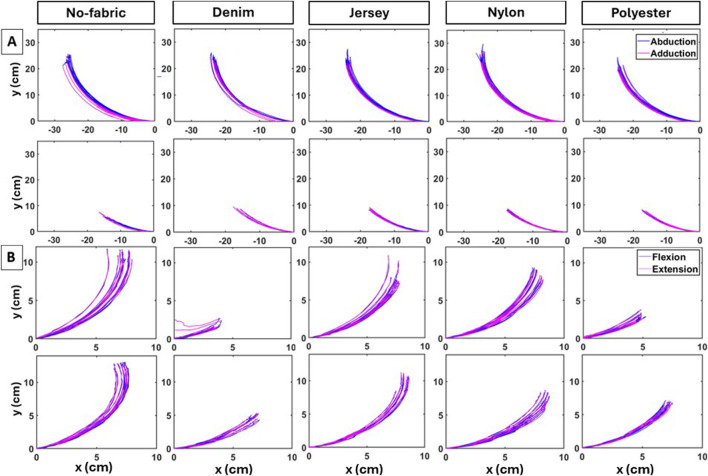
Individual end-effector trajectories of the shoulder **(A)** and elbow **(B)** actuators as the fabric of the actuator enclosures varied. Top- and bottom-row panels in **(A)** were obtained from 1-cell and 2-cell shoulder actuators in S1b configuration, respectively. Similarly, in **(B)**, top- and bottom-row panels were obtained from square and circular elbow actuators in E2 configuration, respectively.

The evolution of the 2D position of the end-effector in the frontal plane (Figure 7A) visually depicts motion variability introduced by each shoulder actuator with the use of different fabric materials for the enclosures. From the respective LSED values shown in Table 4, the variability in actuator performance reduced with the addition of fabric pockets. Overall, the fabric choice for the enclosures significantly influenced the reachable workspace, with some materials increasing and others restricting reachable workspace. In contrast, motion smoothness remained consistent or was even enhanced, depending on the fabric material. Details are provided in the following sections.

**TABLE 4:** LSED values for the obtained trajectories of the shoulder (top) and elbow (bottom) actuators as the fabric of the actuator enclosures varied.

Fabric materials	LSED (Mean ± SD) (cm)			
	**Abduction**		**Adduction**	
	1-cell	2-cell	1-cell	2-cell
No-fabric	1.00±0.28	0.65±0.06	2.11±0.11	1.05±0.08
Denim	0.85±0.20	1.11±0.19	1.56±0.36	1.12±0.06
Jersey	1.00±0.16	0.66±0.04	0.73±0.05	1.30±0.16
Nylon	0.90±0.03	0.62±0.04	1.13±0.02	1.15±0.16
Polyester	0.83±0.10	0.81±0.04	2.52±0.15	1.04±0.08

Fabric materials	**LSED** (Mean ± SD) (cm)			
	**Flexion**		**Extension**	
	Square	Circular	Square	Circular
No-fabric	0.87±0.07	0.71±0.07	5.69±0.72	3.21±0.21
Denim	0.45±0.03	1.25±0.38	0.97±0.13	1.77±0.25
Jersey	0.58±0.10	1.14±0.19	4.22±0.29	3.68±0.16
Nylon	0.60±0.04	0.88±0.23	4.88±0.66	2.65±0.19
Polyester	0.37±0.08	1.43±0.40	1.69±0.13	1.81±0.22

##### 3.2.1.1 Reachable workspace

**FIGURE 8:**
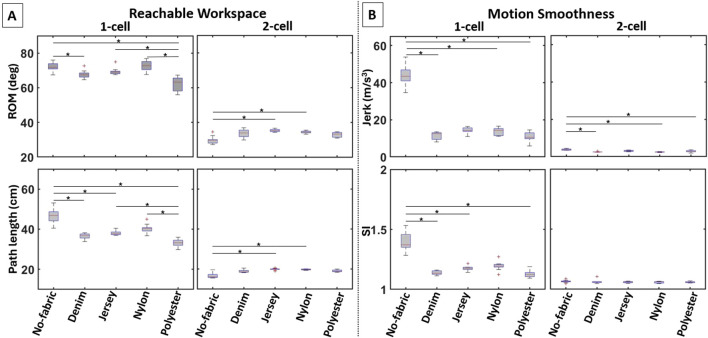
Boxplots of the computed variables for the shoulder actuator variants in terms of **(A)** reachable workspace and **(B)** motion smoothness. The use of different fabrics for the actuator enclosure leads to notable performance differences in most variables.

Varying the fabric properties of the detachable pockets significantly affected shoulder ROM for both the 1-cell ( $\chi^{2}\left(4\right)=33.263$ , p<0.001 ) and 2-cell ( $\chi^{2}\left(4\right)=32.680$ , p<0.001 ) actuators (Figure 8A [top row]). Post-hoc analyses showed that embedding the 1-cell actuator in pockets made of nylon (72.56 ± 2.91^0^, p=1.000 ) and jersey (69.42 ± 2.14^0^, p=0.945 ) did not produce significant changes in the shoulder ROM as compared to non-embedding (72.22 ± 2.55^0^). However, ROM was significantly smaller when polyester (62.27 ± 4.01^0^) and denim (67.72 ± 2.35^0^) were used as opposed to non-embedding (p<0.001) ; actually, values for the polyester were also smaller than those for nylon (p<0.001) and jersey (p=0.022) . In the case of the 2-cell actuator, using jersey (35.50 ± 0.89^0^, p<0.001 ) and nylon (34.52 ± 0.68^0^, p=0.006 ) fabric for the detachable pockets led to a significantly greater ROM than non-embedding (29.42 ± 0.88^0^). The use of polyester (33.02 ± 1.39^0^, p=0.830 ) or denim (33.66 ± 2.22^0^, p=0.078 ) did not affect ROM as compared to non-embedding for this actuator.

Similarly, significant differences in the end-effector’s path length were found for both the 1-cell ( $\chi^{2}\left(4\right)=41.539$ , p<0.001 ) and 2-cell ( $\chi^{2}\left(4\right)=28.855$ , p<0.001 ) actuators (Figure 8A [bottom row]). In the case of the 1-cell actuator, using denim (36.65 ± 1.35 cm, p<0.001 ), polyester (33.21 ± 1.78 cm, p<0.001 ), and jersey (38.12 ± 1.14 cm, p=0.023 ) for the detachable pockets led to shorter path length values than non-embedding (46.81 ± 3.57 cm); actually, values for the polyester were smaller than those for nylon (40.43 ± 2.16 cm, p<0.001 ) and jersey (p=0.037) as well. Nylon (p=1.000) did not affect path length as compared to non-embedding. In contrast, when the 2-cell actuator was embedded in pockets made with nylon (19.80 ± 0.24 cm, p<0.001 ) and jersey (19.93 ± 0.70 cm, p<0.001 ) led to significantly larger path length values as compared to non-embedding (16.76 ± 1.40 cm). Denim (18.87 ± 0.70 cm, p=1.000 ) and polyester (19.08 ± 0.54 cm, p=0.182 ) did not affect path length as compared to non-embedding.

##### 3.2.1.2 Motion smoothness

Significant differences in jerk due to the fabric properties of the detachable pockets were found for both the 1-cell ( $\chi^{2}\left(4\right)=33.251$ , p<0.001 ) and 2-cell ( $\chi^{2}\left(4\right)=33.152$ , p<0.001 ) actuators (Figure 8B [top row]). Embedding the 1-cell actuator in pockets made with denim (11.42 ± 2.11 m^-3^, p<0.001 ), polyester (10.82 ± 2.41 m^-3^, p<0.001 ), and nylon (13.83 ± 1.96 m^-3^, p=0.041 ) significantly reduced jerk as opposed to non-embedding (43.80 ± 5.21 m^-3^). Jersey (14.15 ± 1.79 m^-3^, p=0.091 ) did not affect jerk as compared to non-embedding. Similarly for the 2-cell actuator, using nylon (2.44 ± 0.15 m^-3^, p<0.001 ), polyester (2.78 ± 0.52 m^-3^, p=0.013 ), and denim (2.58 ± 0.20 m^-3^, p<0.001 ) for the pockets led to smaller jerk values than non-embedding (3.80 ± 0.37 m^-3^). Jersey (3.06 ± 0.32 m^-3^, p=0.469 ) did not affect jerk as compared to non-embedding.

Significant differences were also found in SI but for the 1-cell actuator ( $\chi^{2}\left(4\right)=37.590$ , p<0.001 ) only. The SI was smaller when the actuator was embedded in pockets made with jersey (1.18 ± 0.02, p=0.037 ), denim (1.14 ± 0.02, p<0.001 ), and polyester (1.13 ± 0.03, p<0.001 ), but not nylon (1.19 ± 0.04, p=0.274 ), as opposed to non-embedding (1.39 ± 0.08). No significant differences were found for the 2-cell actuator ( $\chi^{2}\left(4\right)=4.178$ , p=0.382 ).

#### 3.2.2 Effects on elbow flexion/extension

The evolution of the 2D position of the end-effector in the sagittal plane demonstrates changes in variability as a result of the fabric material used for the pockets (Figure 7B). As shown in Table 4, LSED values indicate that variability in the trajectories for the square actuator reduced when it was embedded in an enclosure, whereas for the circular actuator, it varied based on the fabric material. Details on the changes in reachable workspace and motion smoothness for each actuator and across fabric type are provided below.

##### 3.2.2.1 Reachable workspace

**FIGURE 9:**
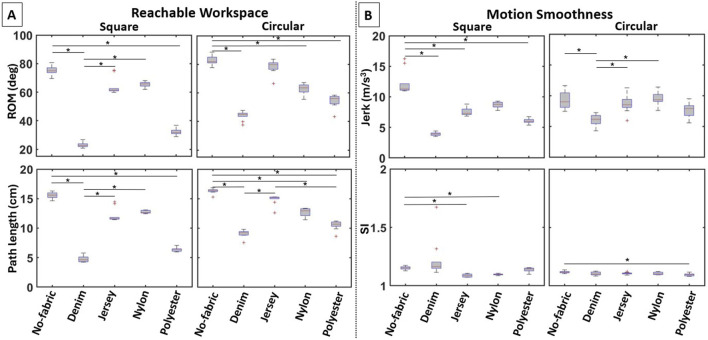
Boxplots of the computed variables for the elbow actuator variants in terms of **(A)** reachable workspace and **(B)** motion smoothness. The use of different fabrics for the actuator enclosure leads to notable performance differences in most variables.

Varying the fabric properties of the detachable pocket significantly varied the elbow ROM for both the square ( $\chi^{2}\left(4\right)=43.717$ , p<0.001 ) and circular $\chi^{2}\left(4\right)=44.199$ , p<0.001 ) actuators (Figure 9A [top row]). Post-hoc analyses revealed that embedding the square actuator in pockets made of denim (23.01 ± 1.65^0^, p<0.001 ) and polyester (32.24 ± 2.24^0^, p<0.001 ) significantly reduced the ROM than non-embedding (75.22 ± 3.75^0^). Using jersey (64.00 ± 5.90^0^, p=0.167 ) and nylon (65.49 ± 1.89^0^, p=0.886 ) did not affect ROM compared to non-embedding. The ROM was also significantly lower when denim was used compared to nylon (65.49 ± 1.89^0^, p<0.001 ) and jersey (64.01 ± 5.90^0^, p=0.004 ). For the circular actuator, ROM was significantly lower when denim (44.08 ± 3.11^0^, p<0.001 ), polyester (44.56 ± 4.60^0^, p<0.001 ), and nylon (62.68 ± 3.81^0^, p=0.039 ) was used, as compared to non-embedding (82.62 ± 3.11^0^). Jersey (78.20 ± 4.79^0^, p=1.000 ) did not affect ROM compared to non-embedding.

Significant differences in the end effector’s path length due to the fabric properties of the pockets were found for both the square ( $\chi^{2}\left(4\right)=45.557$ , p<0.001 ) and the circular ( $\chi^{2}\left(4\right)=45.471$ , p<0.001 ) actuators (Figure 9A [bottom row]). Post-hoc analyses revealed that embedding the square actuator in pockets made of denim (4.75 ± 0.51 cm, p<0.001 ) and polyester (6.39 ± 0.31 cm, p<0.001 ) reduced path length as compared to non-embedding (15.55 ± 0.54 cm). Actually, using denim led to shorter path length than nylon (12.74 ± 0.24^0^, p<0.001 ) and jersey (12.15 ± 1.14^0^, p=0.007 ). For the circular actuator, using denim (9.09 ± 0.64 cm, p<0.001 ), polyester (10.51 ± 0.73 cm, p<0.001 ), and nylon (12.71 ± 0.68 cm, p=0.034 ) fabric for the pockets led to shorter path length as compared to non-embedding (16.32 ± 0.43 cm). Jersey (14.86 ± 0.83 cm) did not affect path length compared to non-embedding and allowed for greater path length as compared to denim (p<0.001) and polyester (p=0.016) .

##### 3.2.2.2 Motion smoothness

Varying the fabric properties of the pockets significantly affected jerk for both the square ( $\chi^{2}\left(4\right)=46.368$ , p<0.001 ) and circular ( $\chi^{2}\left(4\right)=26.567$ , p<0.001 ) actuators (Figure 9B [top row]). Embedding the square actuator in pockets made with denim (3.93 ± 0.28 m^-3^, p<0.001 ), polyester (6.45 ± 0.39 m^-3^, p<0.001 ), and jersey (7.54 ± 0.62 m^-3^, p=0.032 ) significantly reduced jerk as opposed to non-embedding (12.28 ± 1.94 m^-3^). Nylon (8.70 ± 0.49 m^-3^, p=0.976 ) did not affect jerk as compared to non-embedding. For the circular actuator, jerk was significantly lower only when denim (6.02 ± 0.90 m^-3^) was used for the pockets as opposed to non-embedding (9.35 ± 1.50 m^-3^, p<0.001 ). In addition, using denim reduced jerk as compared to nylon (9.51 ± 1.06 m^-3^, p<0.001 ) and jersey (8.67 ± 1.41 m^-3^, p=0.011 ). Polyester did not affect jerk (7.70 ± 1.20 m^-3^, p=0.391 ).

Significant differences in SI were also found for both the square ( $\chi^{2}\left(4\right)=37.184$ , p<0.001 ) and circular ( $\chi^{2}\left(4\right)=12.242$ , p=0.016 ) actuators (Figure 9B [bottom row]). The SI was smaller when the square actuator was embedded in pockets made with jersey (1.01 ± 0.01, p<0.001 ) and nylon (1.10 ± 0.01, p=0.001 ) as compared to non-embedding (1.15 ± 0.01). Denim (1.23 ± 0.17, p=1.000 ) and polyester (1.14 ± 0.02, p=1.000 ) did not affect jerk. When the circular actuator was embedded in pockets only made with polyester (1.10 ± 0.01) the SI was significantly smaller than non-embedding (1.12 ± 0.01, p=0.006 ). There were no significant differences in SI between denim (1.10 ± 0.02, p=0.214 ), jersey (1.11 ± 0.01, p=0.525 ), nylon (1.11 ± 0.01, p=0.736 ), and non-embedding.

## 4 Discussion

Wearable technology for young populations is limited, despite its potential benefits to improve motor function (Arnold et al., 2020; Christy et al., 2016; Henderson et al., 2008). To address this critical gap, our work focuses on developing an UE soft robotic exosuit specifically designed for infants. Textile properties, actuator size and shape, and methods to embed components (like the actuators) onto the exosuit’s substrate are crucial parameters to consider during prototyping and development (Li et al., 2022; Kokubu et al., 2024; Wehner et al., 2013; Zannat et al., 2023). Hence, the focus of this paper was to address the effect of two key features, which pertain to actuator embedding, on the passive substrate of our exosuit prototype: i) the positioning/anchoring of the actuators onto the substrate, and ii) the fabric properties of detachable pockets containing the actuators, for actuators supporting 1-DoF motion about the shoulder (Sahin et al., 2022) and elbow (Sahin et al., 2023) joints. Extensive experiments involving different combinations of actuators, anchoring points, and fabrics for pockets were conducted. The main findings from these experiments confirm that the performance of the actuators can be significantly impacted by variations in anchoring and fabric properties of the pockets. While this result was anticipated, the nature of the change varied considerably, and some interesting trade-offs were revealed. The most appropriate anchoring point was not necessarily the same for all actuator variants, even though they varied in the number or shape of the inflatable cells only. In addition, highly stretchable fabrics not only maintained but even enhanced actuator capabilities, in comparison to the less stretchable materials which hindered actuator performance. Actuator performance was determined by metrics that capture information characterizing actuator function and how much support it can provide to the arms, as well as information relating to more subtle motion characteristics that affect the exosuit’s task support functionality. Specific outcomes are discussed in the following sections.

### 4.1 Trade-off between reachable space and motion smoothness

Our work demonstrated the impact of varying the actuator types and their attachment points and, in addition, it revealed important underlying trade-offs. Specifically, we observed that certain configurations resulting in a larger reachable workspace led to reducing the smoothness of the end-effector’s motion. The types of actuators employed in this work contribute to this trade-off regardless of the anchoring configuration. At the shoulder joint (Figure 3), the 1-cell actuator led to a larger reachable workspace than the 2-cell actuator; however, this benefit was offset by a decrease in motion smoothness. At the elbow joint (Figure 5), the circular elbow actuator led to a larger reachable workspace as compared to the square one; this was again offset by a reduction in motion smoothness. The above observations confirm our prior work on the comparison of different types of actuators (and without the examination of different anchoring points) on the pediatric exosuit (Kokkoni et al., 2020; Sahin et al., 2022; 2023), and the work of others on adult devices (Jarrassé et al., 2010).

The trade-offs between the size of the reachable workspace and motion smoothness irrespectively of actuator anchoring points can be linked to actuator design characteristics. In the case of the 1-cell shoulder actuator, there is a point when there is less amount of air in the actuator’s center as compared to its ends. Then, a small change (increase or decrease) in pressure will result in a sudden flow of air in that middle part of the actuator which in turn will lead to an abrupt motion of the UA thus affecting end-effector motion smoothness. It turns out that embedding the actuator into a pocket (see next section for details) helps with reducing this effect. Furthermore, the two parts of the 2-cell shoulder actuator can overlap with each other when fully inflatable and thus lead to a reduced reachable workspace. This can be remedied by adding a flexible but sturdier jamming component between the two parts to improve the support of the second component (attached to the UA) and thus enable a larger ROM. As for the elbow actuators, by design, the circular shape cells afford a slightly larger expansion than the square ones, for the same critical dimension. This directly leads to larger a reachable workspace for actuators built from circular cells.

When considering the different anchoring points, the aforementioned trade-offs become more convolved. At the shoulder joint, reachable workspace and motion smoothness were affected by varying the points only on the waistline, and not on the UA. This means that the trade-off for the 1-cell is specific to the waistline only, with attaching the actuator along the MAL providing the greatest reachable workspace, but also the least motion smoothness. For the 2-cell actuator, varying the attachment points on both UA and waistline affected the reachable workspace; however, smoothness was affected by varying the points on the UA only. Thus, the trade-off for the 2-cell actuator is specific to anchoring variations on the UA only, with the S2 providing the greatest workspace but also the least smooth motion. At the elbow joint, the trade-off was observed for the square actuator only; anchoring the actuators at an equal distance from the elbow joint provided the greatest reachable workspace but with the least smooth motion.

It becomes evident that careful consideration must be given to the placement of the actuators, taking into account whether a greater reachable workspace or an exceptionally smooth performance is the primary goal. As humans develop and become more proficient in motor skills, they cover a greater workspace and their motion becomes smoother at the same time (Berthier and Keen, 2006; Hogan, 1984; Flash and Hogan, 1985). Typically, a larger reachable workspace is the common goal when developing UE soft wearable technology (Barbosa et al., 2021). This goal, however, may depend on the specific task at hand which may have different requirements. For example, in our population of interest (i.e., infants), the anatomical range of motion has been reported to be between 145° and 170° for the shoulder (Mondal et al., 2022) and between 140° and 155° for the elbow joint (Barad et al., 2013). However, when looking at the task of reaching in midline in this population, the shoulder excursion has been reported to vary on average between 25° and 30°, and for the elbow between 20° and 25° only (Bhat et al., 2007). Thus, although the 2-cell actuator in our exosuit provides a smaller reachable workspace, it can benefit certain actions, such as reaching, by gaining motion smoothness. Thus, trading reachable workspace for smoothness may be more important in some cases. It is worth noting that motion smoothness can be controlled to some extent through the implementation of a suitable feedback controller (Choi et al., 2019) or modulating the percentage of PWM on the pneumatic control board (Sahin et al., 2022), in contrast to the attained reachable workspace which is directly impacted by actuator design parameters (Chen et al., 2023), bounded by limits set by the actuators themselves, and cannot be improved via feedback control.

Additional factors beyond kinematics should be integrated into actuator placement decisions (Lobo et al., 2019). For example, in the case of the 2-cell shoulder actuator, placing it along the AAL was shown to have the largest reachable workspace without losing motion smoothness. One notable drawback associated with placing the actuator along the AAL (or PAL for that matter) is the potential interference it may encounter when the infant is reaching while sitting with support (e.g., on a high chair, booster seat, etc.), which is common before the age of 6 months (Gerber et al., 2010; Adolph and Robinson, 2015). Another example is the decision to place the elbow actuators at the ventral/anterior side of the arm, compared to prior work where actuators were placed on the dorsal/posterior side (Kokkoni et al., 2020; Thalman et al., 2018; Koh et al., 2017). This was necessary to achieve the intended elbow flexion and extension with the type of actuators considered herein. However, this gives another advantage in scenarios where infants’ arms come into contact with surfaces (e.g., armrest of a chair) while in seated or supine positions. In such cases, the actuator’s inflation process might be impeded, leading to performance failure, malfunction of the actuator, and/or discomfort for the infant; which may also lead to safety concerns.

### 4.2 Stretchy-fabric pockets retained/improved actuator performance

This work also shed light into the potential of fabric integration to enhance actuator performance, as assessed by reachable workspace and motion smoothness. Fabric expansion was found to be the most important determinant when noticing changes in the selected variables. Specifically, enclosures made of polyester and denim, the two fabric materials with the least expansion, led to a reduced ROM and path length by half for both cases of shoulder and elbow actuators. However, they also contributed to achieving a smoother end-effector motion. Considering that no fabric negatively affected the smoothness of motion, nylon and jersey were deemed suitable to strike a balance between the reachable workspace and smoothness of motion. While these observations applied at large in our experiments, some variations were observed across the different types of actuators, even enhancing actuator performance in certain cases.

Focusing on the joint level, it appears that the shoulder actuators may benefit more from a careful fabric selection for the enclosures as compared to the elbow actuators. When housed in flexible fabrics like nylon or jersey, the 2-cell actuator resulted in a larger workspace for the end-effector while it was unaffected by the restrictive polyester and denim materials. In contrast, the 1-cell shoulder and elbow actuators did not have a noticeable gain in performance when flexible fabrics were used in an enclosure. Despite this performance improvement, the reachable workspace of the 2-cell actuator remained close to half of that of the 1-cell actuator.

The above observations can be attributed to different reasons related to the type of actuators and mechanics of the shoulder and elbow joints. Regarding the actuators, there are two main differences. From a design viewpoint, shoulder actuators consist of one or two main bladders that inflate, while elbow actuators comprise multiple smaller cells connected in series. When not mounted, a change in the internal pressure of the actuators yields different motions (vertical and linear expansion for the shoulder and elbow actuators, respectively). In addition, the rate of change in actuator shape for the same pressure differential rate is, in general, different owing to the different design. When the actuators are mounted, both actuators are forced to create rotational motion about a single axis, and the above distinctive characteristics can result in the differences observed in our experiments. Furthermore, the difference in the total degrees of freedom between the two joints may play a role as well. Shoulder actuators were more responsive to fabric selection as there was more room for change due to the higher DoF number in the shoulder joint. Although our shoulder actuators act upon 1-DoF, motion about the remaining DoFs is not restricted. Thus, fabrics may produce stabilizing forces to retain and/or enhance the actuator’s performance along its direction of action. Another consideration is the offset of the axis of rotation between the actuator and the targeted joint. Aligning these axes may not be feasible and the offset can vary due to the anatomy of the joints, especially for the shoulder complex (O’Neill et al., 2022). This offset was found to increase further during the operation of both types of actuators. For the elbow actuators, the offset increased during flexion, whereas for the shoulder during abduction. This offset can be the driving force for creating motion about the joint in these directions; thus, the enclosures may reduce that offset depending on the fabrics used. In turn, this modification can also impact subsequent opposing motions, (i.e., elbow extension and shoulder adduction), creating compounded effects.

Previous research on wearable technology has employed a variety of materials with varying degrees of elasticity. For example, neoprene and nylon were used to embed and anchor actuators in an ankle-foot soft robotic orthosis to prevent ankle inversion/eversion (Thalman and Lee, 2020). Coated nylon and nylon-spandex were used for the layered arrangements around pneumatic bladders in a soft wearable glove generating motion about the finger joints to assist in hand opening/closing (Cappello et al., 2018). Also, non-stretch fabric was used for transmitting the contractile force of the actuators to the forearm and performing elbow flexion/extension in another UE exosuit (Park et al., 2022). Neoprene and nylon have also been used in the base layer of shoulder exosuits (Natividad et al., 2020; Lessard et al., 2018), while cotton and nylon (of the same composition as ours) were used for the substrate surrounding the body and the shoulder joint for the design of UE exosuit (Golgouneh et al., 2021). As far are pediatric wearables are concerned, materials such as vinyl have been used for the underarm casings housing wire bundles producing the necessary forces to elevate the arms as well as nylon webbing for the belt and wrist straps to stabilize the supports (Hall and Lobo, 2018), while a pneumatic bladder made of TPU-coated nylon taffeta was embedded on a stretch shirt (95% cotton, 5% spandex) using a piece of cover fabric (performance nylon spandex power mesh) (Li et al., 2019). It is apparent from the above that, although many works have considered nylon-based fabrics, some changes in the ratio of nylon to other materials, the weaving process, and any other processes like coating may yield fabrics with completely different properties that may, in turn, cause various effects on the exosuits. A tighter integration with textile engineering could thus help push forward the state of exosuits.

### 4.3 Strengths and limitations

In this work, we treated the shoulder and elbow actuators separately. This allowed us to assess the effect of different parameters such as anchoring and fabric properties of the enclosures consistently and systematically, to best determine which configurations may lead to improved performance. However, the complete support afforded by our exosuit is a fusion of the motion generated by the elbow and shoulder actuator. Cross-actuator compounded effects on end-effector motion can be complex and still need to be studied and modeled. Yet, an understanding of each actuator’s characteristics can help discover such compounded effects. Ongoing work focuses on this direction.

The evaluation was conducted using a custom-designed physical model matching the dimensions of a one-year-old infant. However, it is important to acknowledge that certain important features are not captured well with a model. Most crucially, actual joint dynamics can be different between an engineered device and an infant, while the motion of the infant (which from a mathematical modeling standpoint can be viewed as an exogenous input to the exosuit) is not captured within a physical model. While human subject testing is a critical component to be addressed in future work, further assessment with the engineered model will ensure the exosuit is safe for testing with infants.

Lastly yet importantly, the findings of this work can serve as the basis to introduce kinematic and dynamic models of arm motion and force control for UE exosuits. Force control is the basis for offering assistive, as-needed feedback to the user. To be able to determine satisfactory (and safe) control effort, it is important to first understand appropriate features about how to integrate the actuators onto a passive substrate, and then, for those viable configurations, model their motion and create forcing profiles. The latter is part of ongoing research enabled by this present research effort.

## 5 Conclusion

We investigated how different configurations pertaining to shoulder and elbow actuator embedding on the passive substrate of a pediatric exosuit for UE motion assistance can affect key performance metrics. The configurations studied in this work varied based on actuator anchoring and the type of fabric used in actuator enclosures. Shoulder adduction/abduction and elbow flexion/extension using two similar but distinct actuators for each case were treated separately. The considered metrics were grouped into two categories; reachable workspace, which included joint ROM and end-effector path length; and motion smoothness, which included end-effector path SI and jerk. The former category aimed to capture first-order terms (i.e., rotations and displacements) that capture overall gross motion, while the latter category aimed to shed light on differential terms that correlate with the quality of the attained motion. Extensive experimentation was conducted for each individual considered configuration, and statistical analyses were used to establish distinctive strengths, weaknesses, and trade-offs among those configurations. The main findings from experiments confirm that the performance of the actuators can be significantly impacted by variations in the anchoring and fabric properties of the enclosures while establishing interesting trade-offs. Specifically, the most appropriate anchoring point was not necessarily the same for all actuator variants. In addition, highly stretchable fabrics not only maintained but even enhanced actuator capabilities, in comparison to the less stretchable materials which turned out to hinder actuator performance. We anticipate that the established trade-offs can serve as guiding principles for other researchers and practitioners developing UE exosuits. In addition, the findings from individual actuator assessments help propel forward ongoing work focusing on the study of compounded actuator motion as well as force feedbackcontrol design.

## Data Availability

The original contributions presented in the study are included in the article. Supplementary videos can be found here. Further inquiries can be directed to the corresponding author.

## Footnotes

1 Passive substrate refers to the fabric material placed on the body of the physical model to act as the primary support structure for attaching further elements (Sanchez et al., 2021), such as the actuators in this work.
